# Genome-wide analysis of transcription factors during somatic embryogenesis in banana (*Musa* spp.) cv. Grand Naine

**DOI:** 10.1371/journal.pone.0182242

**Published:** 2017-08-10

**Authors:** Shivani, Praveen Awasthi, Vikrant Sharma, Navjot Kaur, Navneet Kaur, Pankaj Pandey, Siddharth Tiwari

**Affiliations:** 1 National Agri-Food Biotechnology Institute (NABI), Department of Biotechnology, Ministry of Science and Technology (Government of India), Knowledge City, Mohali, Punjab, India; 2 Department of Biotechnology, Panjab University, Chandigarh, India; Bhabha Atomic Research Centre, INDIA

## Abstract

Transcription factors *BABY BOOM (BBM)*, *WUSCHEL (WUS)*, *BSD*, *LEAFY COTYLEDON (LEC)*, *LEAFY COTYLEDON LIKE (LIL)*, *VIVIPAROUS1 (VP1)*, *CUP SHAPED COTYLEDONS (CUC)*, *BOLITA (BOL)*, and *AGAMOUS LIKE (AGL)* play a crucial role in somatic embryogenesis. In this study, we identified eighteen genes of these nine transcription factors families from the banana genome database. All genes were analyzed for their structural features, subcellular, and chromosomal localization. Protein sequence analysis indicated the presence of characteristic conserved domains in these transcription factors. Phylogenetic analysis revealed close evolutionary relationship among most transcription factors of various monocots. The expression patterns of eighteen genes in embryogenic callus containing somatic embryos (precisely isolated by Laser Capture Microdissection), non-embryogenic callus, and cell suspension cultures of banana cultivar Grand Naine were analyzed. The application of 2, 4-dichlorophenoxyacetic acid (2, 4-D) in the callus induction medium enhanced the expression of *MaBBM1*, *MaBBM2*, *MaWUS2*, and *MaVP1* in the embryogenic callus. It suggested 2, 4-D acts as an inducer for the expression of these genes. The higher expression of *MaBBM2* and *MaWUS2* in embryogenic cell suspension (ECS) as compared to non-embryogenic cells suspension (NECS), suggested that these genes may play a crucial role in banana somatic embryogenesis. *MaVP1* showed higher expression in both ECS and NECS, whereas *MaLEC2* expression was significantly higher in NECS. It suggests that *MaLEC2* has a role in the development of non-embryogenic cells. We postulate that *MaBBM2* and *MaWUS2* can be served as promising molecular markers for the embryogencity in banana.

## Introduction

**Fig 1:**
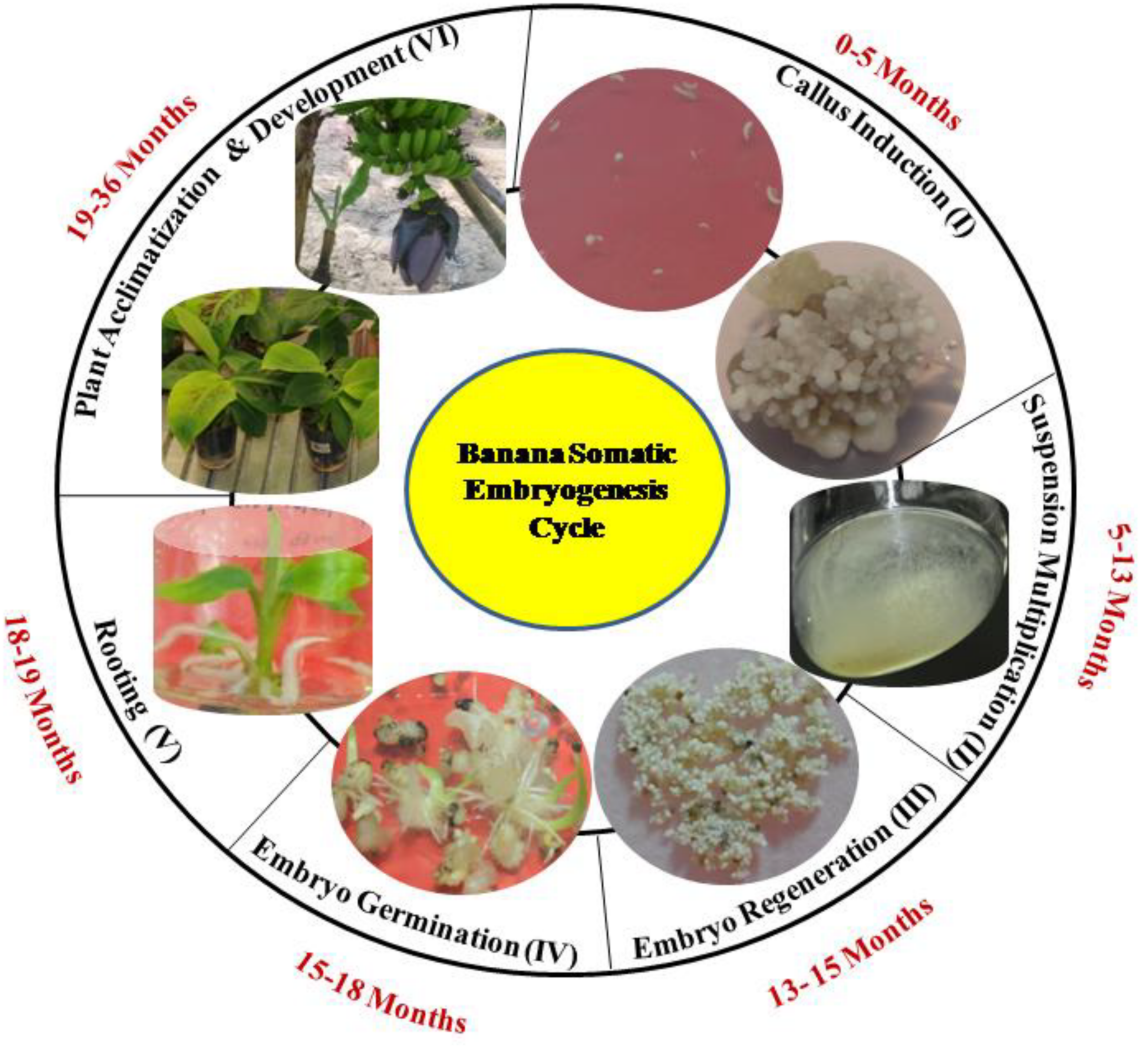
Banana somatic embryogenesis developmental stages. Different developmental stages of banana somatic embryogenesis cycle are following: (I) Callus Induction (0–5 months) (II) Suspension and Multiplication (5–13 months) (III) Embryo Regeneration (13–15 months) (IV) Embryo Germination (15–18 months) (V) Rooting (18–19 months) (VI) Plant Acclimatization and development (19–36 months).

Banana is an important staple food fruit crop in several developing countries. Being a vegetatively propagated plant, its multiplication index through sucker is very low [1]. Somatic embryogenesis (SE) is considered to be an important route for mass production [2–4] and development of transgenic [5–6] banana plants. SE through embryogenic cell suspension (ECS) has been reported in various banana cultivars [7–9]. The process includes different developmental stages viz., callus, ECS, regeneration, germination, rooting and acclimatization (Fig 1). The occurrence of somaclonal variation in SE through callus culture has been demonstrated in several plant species [10–12]. A callus phase in tissue culture, the use of growth regulators, the number and duration of subcultures, stress and the genotype are the factors that enhance the somaclonal variation and restrict the potential use of SE [11–12]. Therefore, the molecular factors which are known to regulate SE in other plants can provide a lead to understand the mechanism of SE in banana.

Transcription factors (TFs) are the evolutionary conserved regulatory proteins, which play a significant role in transcriptional regulation of gene expression. Plant TFs reported to have a higher rate of lineage-specific expansion [13–14]. They are imperative molecules in the regulation of different biological processes, including SE in plants. Certain TFs such as *BABYBOOM* (*BBM*) [15–19], *WUSCHEL* (*WUS*) [20–23], *BSD* [24–25], *LEAFY COTYLEDON* (*LEC*) [26–27], *LEAFY COTYLEDON LIKE* (*LIL*) [28–29], *VIVIPAROUS1* (*VP1*) [30–31], *BOLITA* (*BOL*) [32] and *AGAMOUS-LIKE* (*AGL*) [33] are highly specific to plant lineage, suggesting their importance in plant-specific processes. These TFs are restricted to plant lineage and characterized by the presence of conserved domain in their proteins. The study directing towards understanding the role of these TFs in banana could be of great interest and has the potential for improvement of SE. For instance, *BBM* expression patterns in cacao tissue reported as a biomarker for embryogenesis [19]. *WUS* play a vital role in SE by promoting the vegetative to embryogenic transition in arabidopsis [21]. *BSD* is known to be associated with cell proliferation during the SE [24–25, 34]. *LEC* [27] and *L1L* [28] contain HAP3 subunit and reported for their role in embryo development, morphogenesis, and cellular differentiation. The role of *AGL* has been demonstrated in SE of arabidopsis and radish plants [35–36].

Several other TFs are known to be involved in the process of only organogenesis or both SE and organogenesis. For example, *VP1* regulates seed dormancy and involved in organogenesis and SE [30, 37–38]. However, *CUC* plays a role only in the organogenesis [39–40]. *CUC* is also known to promote adventitious root formation in calli [41]. Another TF like *BOL* is known to be involved in the regulation of the cell expansion and proliferation [32].

Earlier, efforts have been made to characterize different TFs for their role in SE in several plant species [15, 20, 24, 28], but no report is available in banana. In the present study, 18 genes belonging to the nine TF families were identified in the banana genome. Phylogenetic tree analyses of these genes in land plants including monocot, dicot, and gymnosperm were conducted for their molecular evolution. In addition, the expression patterns of these genes were characterized during the critical steps of *in-vitro* cultures of cv. Grand Naine (AAA).

## Materials and methods

### Identification, chromosome distribution and exon-intron prediction of TFs in banana

Nine TF families (*BBM*, *WUS*, *BSD*, *LEC*, *L1L*, *VP1*, *CUC*, *BOL* and *AGL*) were selected for the *in-silico* study. Homologs of these TFs were identified in arabidopsis, maize and rice (TAIR, http://www.arabidopsis.org; http://bioinformatics.psb.ugent.be/plaza; TIGR, http://rice.plantbiology.msu.edu) and used as a query sequence in BLASTP search to retrieve the sequences from the banana genome (http://banana-genome.cirad.fr/). The putative protein sequences resulting from each blast search (E- value 10^−5^) in banana were collected and redundant sequences were removed. Gene models were refined and genomic coding sequences (CDS) were retrieved from the banana genome hub database [42, http://banana-genome.cirad.fr/]. For chromosomal localization, genomic coordinates were recovered from the banana genome. Genomic sequences of the selected banana TFs were downloaded from Gramene database [43, http://www.gramene.org/]. Genomic and CDS sequences of all homologs were aligned using multialin software [44, multalin.toulouse.inra.fr/multalin/]. The number of exons and introns were calculated manually.

### Phylogenetic analysis, motif identification and sequence analysis

Protein sequences of the selected TFs of banana and their homologs from angiosperm (monocot and dicot) and gymnosperm were used for phylogeny analyses. The phylogenetic tree was constructed using the neighbor-joining method with MEGA 6.0 software [45]. The unrooted tree was generated through 1000 bootstrap values for the reliability of the tree. Multiple sequence alignment was carried out by using CLC genomic workbench (QIAGEN Denmark). The amino acid sequence corresponding to TF families in banana were studied for conserved domains analysis using conserved domain database [46]. Theoretical isoelectric point (pI) and molecular weight (MW) were predicted using the Compute pI/MW tool on the ExPASy server [47, http://web.expasy.org/compute_pi/]. Transmembrane helix (TMH) was analyzed on the TMHMM server v 2.0 [48, http://www.cbs.dtu.dk/services/TMHMM-2.0/] and the subcellular localization of deduced proteins was predicted on the Target P 1.1 server [49, http://www.cbs.dtu.dk/services/TargetP/].

### Callus development

Total seventy immature male flower buds (source of explant) of cv. Grand Naine were collected from the experimental field of National Agri-Food Biotechnology Institute (NABI), Mohali, Punjab, India (310 m above sea level; Latitude 30° 47’ North; Longitude 76° 41’ East). Immature male flowers (explants) of rank (1–15) adjacent to the floral apex were isolated and inoculated on callus induction medium for callus formation [7, 50]. Six hundred explants derived from forty flower buds were inoculated on 2, 4-dichlorophenoxyacetic acid (2, 4-D) containing medium, while the four hundred fifty explants prepared from thirty buds were cultured on 2, 4-D -free MS basal medium. All cultures were incubated under the dark condition at 27°C for the emergence of somatic embryos in plant tissue culture chambers (Percival, USA). After 12 Week (W) of incubation, embryogenic calli (with proembryos) and non-embryogenic calli were identified under the upright microscope (Leica Microsystems, Germany), collected, frozen immediately in the liquid nitrogen and stored at −80°C until further use.

### ECS establishment

Nearly six months (24W) old embryogenic calli comprised with somatic embryos and non-embryogenic calli were inoculated in suspension medium containing 2, 4-D and zeatin [7]. Suspension culture was kept in dark at 27°C with agitation at 90 rotations per minute (Kuhner, Switzerland) and sub-cultured weekly. ECS samples were collected at 0W, 1W, and 24W intervals while the NECS collected at 0W, and 1W for further study.

### Histological analysis

Samples collected from callus induction medium with 2, 4-D and without 2, 4-D were used for histological analysis. Samples were preserved in 70% ethanol at 4°C for one day [51] and then fixed in blocks using freezing solution (Leica Biosystems, Germany). Ten to twelve μm thin sections were prepared by using cryomicrotome (Leica Biosystems, Germany) and observed under the microscope (Leica Microsystems, Germany). For suspension culture analysis, cells were placed on the slide along with suspension medium and cover slip. The embryogenic and non-embryogenic cells were stained with iodine to confirm the presence of starch granules. These cells were observed directly under the upright microscope (Leica Microsystems, Germany).

### Laser capture microdissection (LCM) of embryogenic cells

RNAase free conditions (tools, solutions, handling) were followed throughout the experiment. The experiment was performed as per previously described protocol [52]. In brief, banana callus (24W) containing embryos were fixed at -23°C under vacuum using tissue freezing medium (Leica biosystems, Germany). Tissue blocks were fixed with the holding clamp of cryomicrotome (Leica biosystems, Germany) and 10–12 μm thin sections were prepared. These sections were taken on slides, air-dried at room temperature and observed under LCM microscope (Zeiss, Germany).

For microdissection, embryos were identified and marked using PALM (Zeiss, Germany) tool. Tissues were snipped-off with a laser beam along with marking in RNase-free tubes and stored at -80°C for RNA isolation.

### RNA isolation and cDNA synthesis

Total RNA was isolated from different samples using RNA extraction kit (Sigma-Aldrich, USA). Isolated RNA was treated with DNase I kit (AmbionThermo Scientific, USA) to eliminate DNA contamination. Total RNA was analyzed by agarose gel electrophoresis for size and integrity. The quantification of total RNA was done with a NanoQuant (Infinite 200 PRO NanoQuant, Austria). DNA-free RNA was used for cDNA first strand synthesis by using revert aid first strand cDNA synthesis kit (Thermo Scientific, USA) as per manufacturer’s protocol. Oligo dT primer was used for cDNA preparation.

### Quantitative real-time PCR (qPCR)

The qPCR study was performed using ABI 7700 Sequence Detector (Applied Biosystems, USA). Housekeeping gene *Actin1* (GenBank Accession No. AF246288) was used to normalize variant expression of selected genes [53–54]. The primers were tested for single band amplification using conventional end-point PCR. Melting curve study was carried out using qPCR. The total volume of each reaction was 10 μl and consisted of 1X SYBR Green Master mix (Applied Biosystems, USA) 5 pmol of each primer, 0.5 μl cDNA template and sterile distilled H_2_O. PCR conditions followed during real-time PCR experiment were: step (1) 50°C 2 min, step (2) 95°C 10 min, step (3) (95°C 0.15 min, 60°C 1 min) x 40 cycles, followed by the thermal dissociation curve. The relative expression level was analyzed using the 2^-ΔΔCt^ method [55], where ΔΔCt = (Ct target—Ct actin) time _x_−(Ct target—Ct actin) time _0_. Primer details are mentioned in S1 Table. All experiments were performed in biological triplicates and each experiment consisted of three technical replicates. The t-test was carried out for assessment of statistical significance of data.

## Results

### Identification and sequence analysis of SE related TFs in *Musa acuminata*

The BLAST search was carried out for identification of BBM, WUS, BSD, LEC, L1L, VP1, CUC, BOL, and AGL protein sequences in banana by using query sequences of arabidopsis (27), rice (8), and maize (13). A total of 18 sequences belonging to nine TF families were retrieved from the banana genome. All deduced protein sequences contain conserved domains of their respective TF families (S1, S2, S3, S4, S5, S6, S7 and S8 Figs). Protein sequence identity between banana TFs and their homologs in arabidopsis, maize, and rice ranges from 24% to 83%, 25% to 74%, and 45% to 74%, respectively (S2 Table). Various features such as locus ids, chromosomal coordinates, predicted protein MW, pI, subcellular localization and TMH of banana sequences were summarized in Table 1. Protein length and MW of all TFs ranged from 63 to 678 amino acids and 7.20 to 75.04 kDa, respectively. Most of the TFs (11) showed pI in acidic range (4.3–6.83), while others had an alkaline range (8.6–10.14). However, only *MaLEC*1 had shown neutral pI. *MaBBM1*, *MaBBM2*, and *MaLIL1* were localized in the chloroplast, whereas *MaBSD3* and *MaAGL1* in mitochondria. Subcellular localization of other TFs was not available. No transmembrane domain was predicted in any TF.

**Table 1:** Structural features of SE related transcription factors in *Musa acuminata*.

Gene Name	Gene ID	Chromosome Position	Coordinates	Protein Length	Predicted pI	MW (kDa)	Subcellular Localization	Trans Membrane Helix (TMH)
*MaBBM1*	GSMUA_Achr3P21460_001	3	22424668:22429036	612	6.30	66.8	Chloroplast	0
*MaBBM2*	GSMUA_Achr2P05880_001	2	10811480:10815136	572	6.83	62.8	Chloroplast	0
*MaWUS1*	GSMUA_Achr8P02040_001	8	1508245:1509831	128	9.8	15.02	-	0
*MaWUS2*	GSMUA_Achr10P26570_001	10	30070543:30072984	233	8.9	25.7	-	0
*MaBSD1*	GSMUA_Achr6P00640_001	6	420148:428830	421	4.3	46.1	-	0
*MaBSD2*	GSMUA_Achr8P25810_001	8	29568544:29574681	295	4.8	33.1	-	0
*MaBSD3*	GSMUA_Achr2P17230_001	2	17835096:17843511	496	4.3	54.9	Mitochondria	0
*MaLEC1*	GSMUA_Achr10P12560_001	10	21559530:21570493	482	7.0	54.6	-	0
*MaLEC2*	GSMUA_Achr3P23760_001	3	24489817:24495395	368	6.4	41.82	-	0
*MaLIL1*	GSMUA_Achr4P26330_001	4	25686876:25690660	269	5.3	30.2	Chloroplast	0
*MaLIL2*	GSMUA_Achr11P06580_001	11	5174935:5181575	205	5.6	25.8	-	0
*MaVP1*	GSMUA_AchrUn_randomP18520_001	random	88731276:88735269	678	6.2	75.04	-	0
*MaCUC1*	GSMUA_Achr9P20090_001	9	23156505:23159553	317	5.3	35.7	-	0
*MaCUC2*	GSMUA_Achr9P00570_001	9	481453:483257	168	9.3	19.5	-	0
*MaCUC3*	GSMUA_Achr10P22350_001	10	27580397:27583307	355	8.6	38.4	-	0
*MaBOL*	GSMUA_Achr6P19570_001	6	13852244:13854193	89	6.4	9.7	-	0
*MaAGL1*	GSMUA_Achr5P20280_001	5	22027677:22029271	63	10.14	7.2	Mitochondria	0
*MaAGL2*	GSMUA_Achr8P07230_001	8	4776954:4778864	236	9.4	25.8	-	0

### Chromosome localization and exon-intron composition

**Fig 2:**
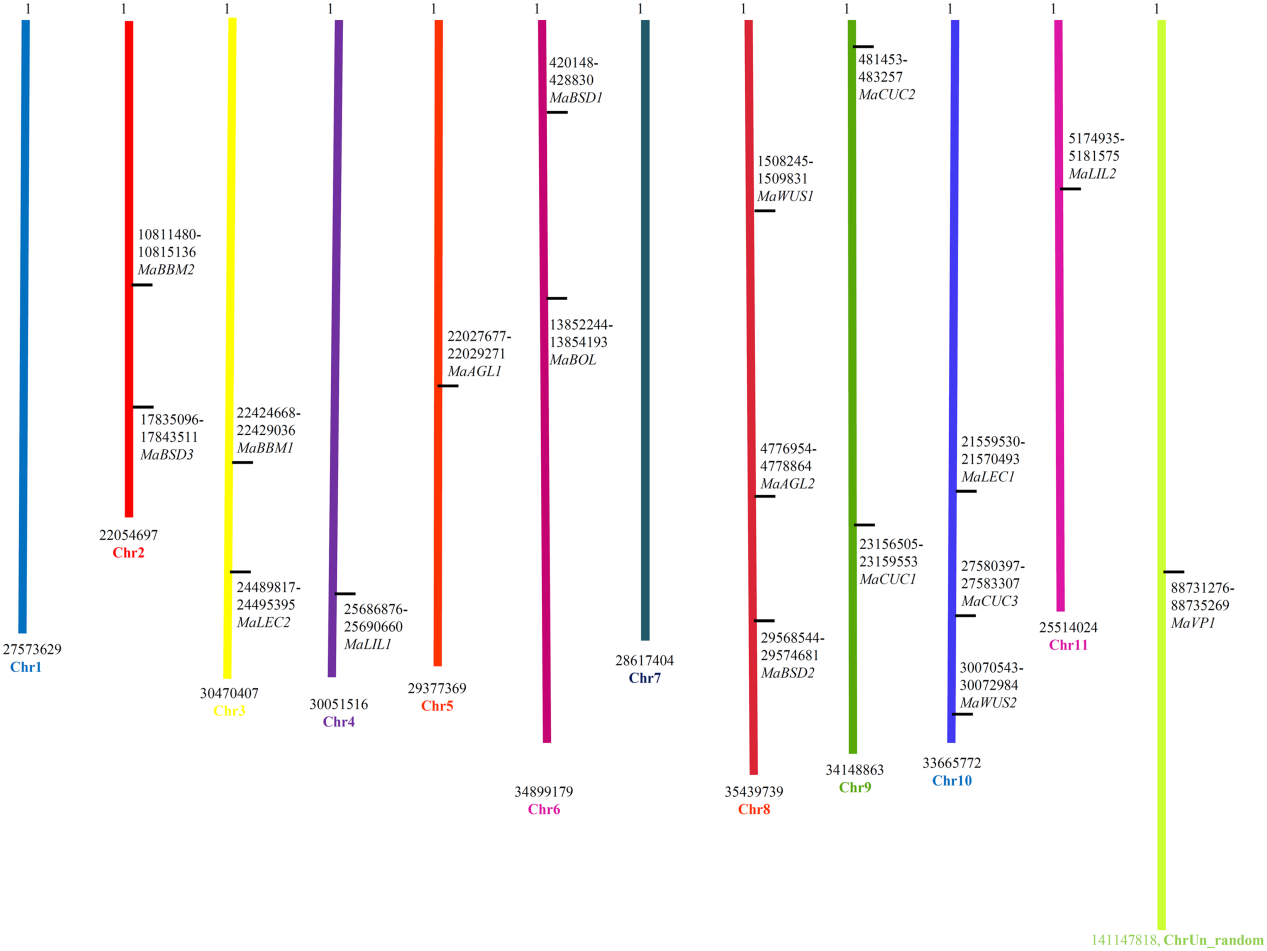
Localization of transcription factors and genes on banana chromosomes. The black vertical lines on the chromosomes indicate the positions of the respective genes. Numbers represent nucleotide base pair positions with the name of the respective gene.

All TFs except one (*MaVP1*) that was found to be associated with un_random chromosome could be mapped on banana chromosomes (Fig 2). All the mapped TFs were distributed over nine chromosomes {*MaAGL1* (Chr5), *MaAGL2* (Chr8), *MaBBM1* (Chr3), *MaBBM2* (Chr2), *MaBSD1* (Chr6), *MaBSD2* (Chr8), *MaBSD3* (Chr2), *MaL1L1* (Chr4), *MaL1L2* (Chr11), *MaWUS1* (Chr8), *MaWUS2* (Chr10), *MaCUC1* (Chr9), *MaCUC2* (Chr9), *MaCUC3* (Chr10), *MaLEC1* (Chr10), *MaLEC2* (Chr3) and MaBOL (Chr6)}.

*MaAGL1*, *MaAGL2*, and *MaWUS1* were intron-free genes, while other TFs have shown the presence of introns varying from 1 to 11. *MaBBM1*contained the highest number of introns (11) while its homolog in arabidopsis has only 8 introns. Except for *BOL* and *CUC*, no other TFs were found to have its homologs in rice (S3 Table).

### Phylogenetic study and conserved motif analysis

**Fig 3:**
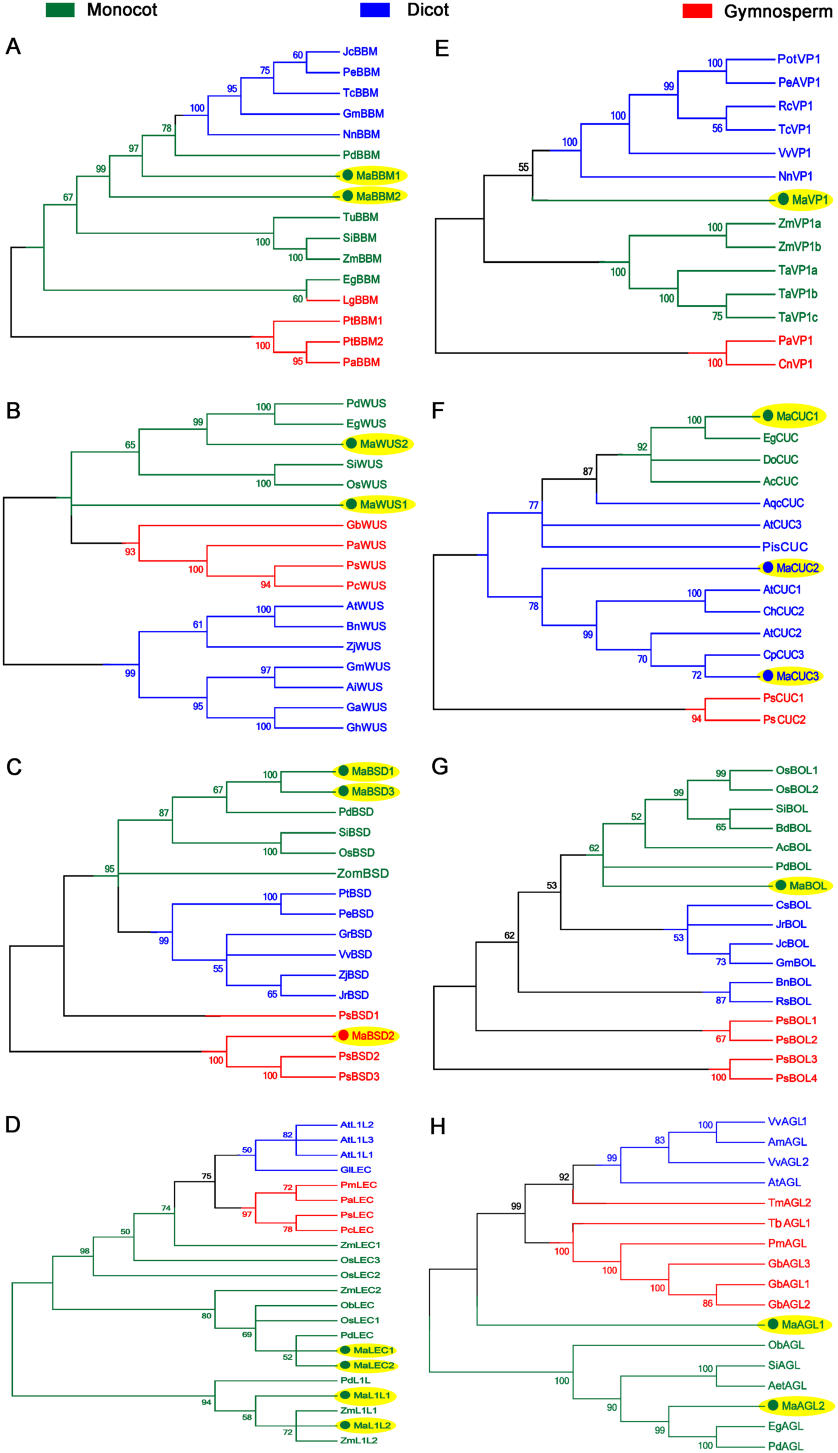
The phylogenetic trees of transcription factors are showing clustering of sequences with different taxonomic groups of plants. (A) Integration of BBM TFs within AP2/ERF family members from the different taxonomic group of plants. (B) Integration of WUS TFs with other WOX gene family members from the different taxonomic group of plants. (C) Integration of BSD TFs with its other family members from the different taxonomic group of plants. (D) Integration of LEC/L1L TFs with other CBF family members from the different taxonomic group of plants. (E) Integration of VP1 TFs with its other homologs (ARF family) from a different taxonomic group of plants. (F) Integration of CUC TFs within NAC family members from the different taxonomic group of plants. (G) Integration of BOL TFs with its other homologs (BOL A family) from a different taxonomic group of plants. (H) Integration of AGL TFs with its other homologs (MADS family) from a different taxonomic group of plants.

The TF sequences from the gymnosperm, monocot and dicot were included in the phylogenetic tree construction. As expected, all the homologs of banana TFs were clustered with the monocot clade except MaBSD2, MaCUC2, and MaCUC3. MaBSD2 grouped with gymnosperm, whereas MaCUC2 and MaCUC3 clustered with dicot (Fig 3).

The amino acid sequences corresponding to 9 TFs in banana were studied for conserved domains analysis (S1, S2, S3, S4, S5, S6, S7 and S8 Figs). MaBBM was found to have DNA binding sites within two conserved AP2 domains. It is required for transcription regulation of developmental processes, whereas MaWUS had DNA binding site along with specific DNA base contact site in the homeobox conserved domain. It is known to play a key role in plant development. MaBSD protein containing signature BSD domain is synapse-associated protein and is not much explored. LEC/L1L was noticed to contain a conserved HAP3 domain that played an important role in signal transduction and light harvesting. MaVP1 contained one acidic amino-terminal region (A1) and three basic regions (B1, B2, B3), having a role in seed development and auxin transport. MaBOL possessed BolA region that is involved in callus induction. MaCUC a member of NAC gene family is known to be related with organogenesis. The presence of these domains in the selected TFs confirmed their belongings to the respective families.

### Development of somatic embryogenesis

**Fig 4:**
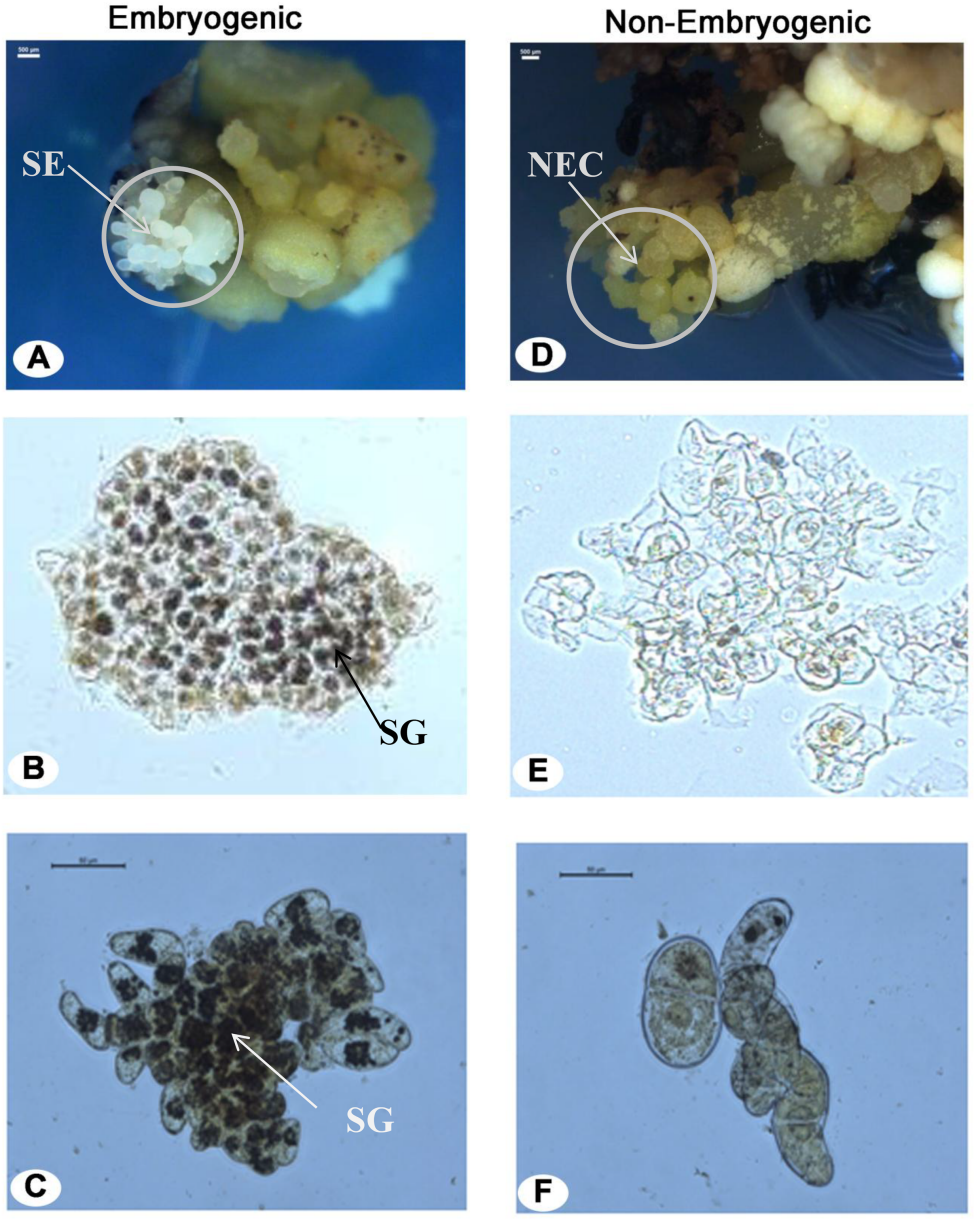
Histological analysis of callus and cell suspension cultures under the microscope. (A) Somatic embryos in callus. Iodine-stained (B) embryogenic cells and (C) ECS with packed starch granules in deep black color. Iodine-stained (D) non-embryogenic callus and(E) NECS. SE, NEC, and SG in the figure represent somatic embryos, non-embryogenic callus, and starch granules, respectively.

Total eighteen explants responded on the 2, 4-D containing medium with 3% frequency of embryogenic callus formation. On the other side, none of the explants responded for embryogenecity on MS basal medium (without 2, 4-D). Nearly after 3–6 months (12W-24W) of incubation one or few somatic embryos emerged on the surface of callus. Microscopic visualization of callus consisted of somatic embryos, non-embryogenic callus, and their respective suspension cells are shown in Fig 4. The somatic embryos were globular and translucent in nature and present on the surface of the callus (Fig 4A). Embryogenic region beneath the somatic embryos contained many numbers of friable embryogenic cells and suitable for initiation of ECS. The non-embryogenic callus was yellow, compact, hard and lacked embryo-like structure on the surface (Fig 4D). Embryogenic as well as non-embryogenic cells were inoculated in the suspension medium. Suspension cultures of independent embryogenic and non-embryogenic lines were kept separately in the shaker. Embryogenic cell aggregates multiplied and formed many lobed structures from peripheries of which new aggregates were released. Non-embryogenic cells did not divide and eventually led to cell death after 1W. The responded ECS were sub-cultured weekly and maintained for more than 20 months with the high efficiency of embryogenic response.

**Fig 5:**
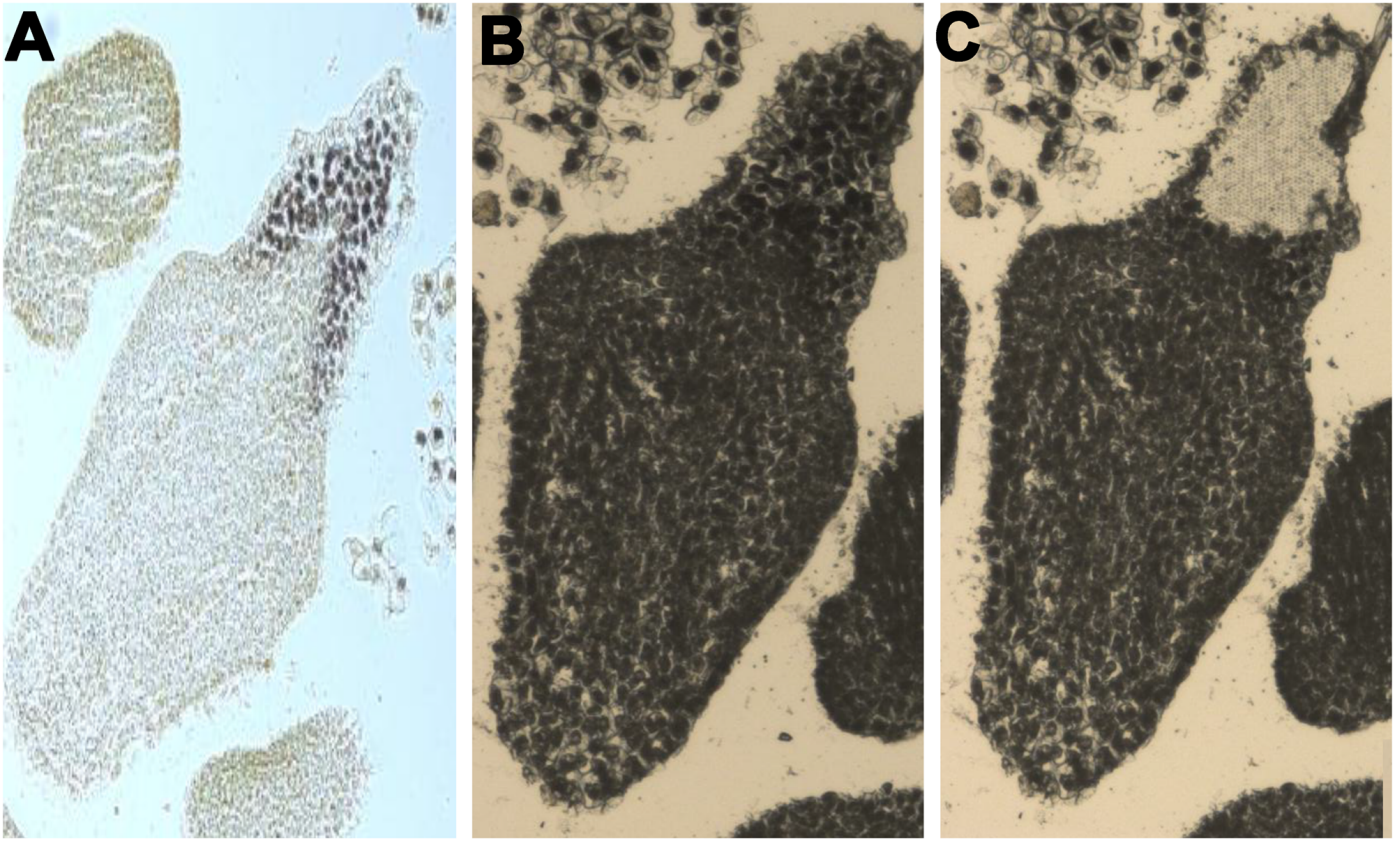
Representative image of LCM-derived tissue for RNA isolation. (A) Iodine-stained embryogenic cells for LCM. (B) Somatic embryo before LCM-derived tissue extraction. (C) Somatic embryo after embryogenic cells snipped off.

Embryogenic cells have dense cytoplasm with small vacuole and abundant starch granules at borders (Fig 4B). These cells were mostly isodiametric and spherical in shape. The cells of non-embryogenic nature were highly vacuolated, contained fewer starch granules and abnormal in shape and size (Fig 4E). These observations are in agreement with previous studies [2, 7, 56]. The darkly stained cell aggregates have also been confirmed the presence of dense cytoplasm with abundant starch granules in ECS (Fig 4C). The similar observation was reported in the other studies [2, 57]. These embryogenic cells were spherical in shape and multiplied at a higher rate. The cells in NECS were the irregular shape, highly vacuolated and hardly contained any starch granules (Fig 4F). Apart from this, the potent embryogenic cells were snipped off using LCM from a mature embryo, which is used as 0W ECS for the expression study (Fig 5).

### Expression study of TFs in response to 2, 4-D treatment in callus

**Fig 6:**
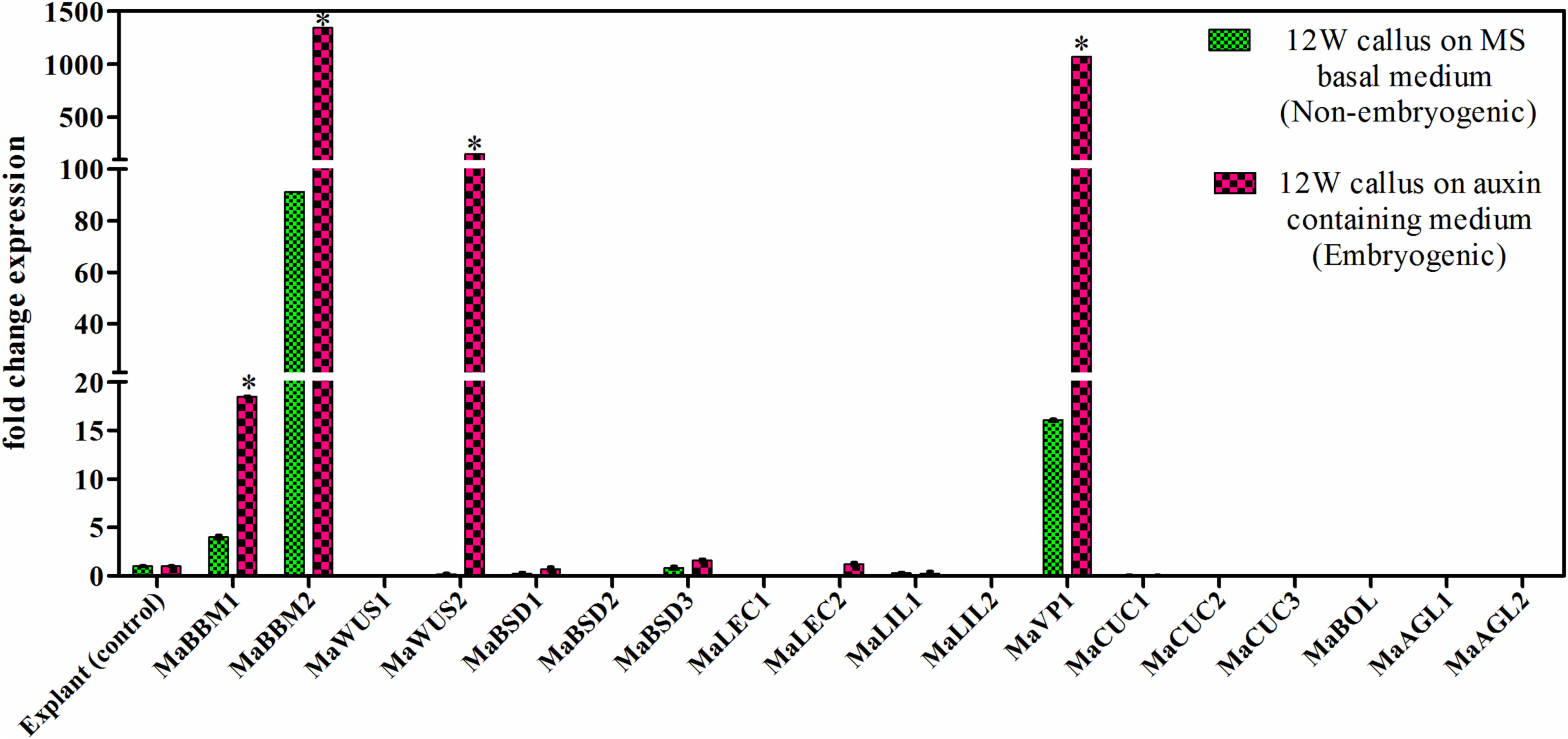
Quantitative real-time PCR study of selected TFs in callus. The differential expression of TFs in 12W callus is shown in response to 2, 4 –D treatment. The gene expression was normalized using *Actin1* as an internal control. Bars denote mean fold expression as compared to the control (explant) ± SD. Statistical analysis was performed using student’s paired t-test. Statistical significance was checked at p<0.05 (*) with respect to the MS basal medium (without 2, 4 -D treatment).

Banana male flowers were inoculated on callus induction medium without 2, 4-D and with 2, 4-D treatment. After 12W, the callus was harvested and further used for gene expression study (Fig 6). The explant (male flower) was used as a control for the study. The expression of *MaBBM1*, *MaBBM2*, and *MaVP1* was upregulated on 2, 4-D free medium with respect to the explant. The significantly higher expression of *MaWUS2* along with *MaBBM1*, *MaBBM2*, and *MaVP1* was observed on 2, 4-D supplemented medium. The expression of *MaBBM1*, *MaBBM2*, and *MaVP1* was increased to 4.6, 14.7, and 66.9 fold respectively, in 2, 4-D supplemented medium as compared to 2, 4-D free medium (Fig 6). *MaWUS1*, *MaBSD1*, *MaBSD2*, *MaBSD3*, *MaLEC1*, *MaLEC2*, *MaL1L1*, and *MaCUC1* showed no significant change (p<0.05) in their expression level in both conditions (with 2, 4-D and without 2, 4-D) as compared to the explant. The expression of *MaCUC2*, *MaCUC3*, *MaBOL*, *MaAGL1*, and *MaAGL2* was not detected in the study.

### Gene expression analysis of TFs at different developmental stages of ECS and NECS

**Fig 7:**
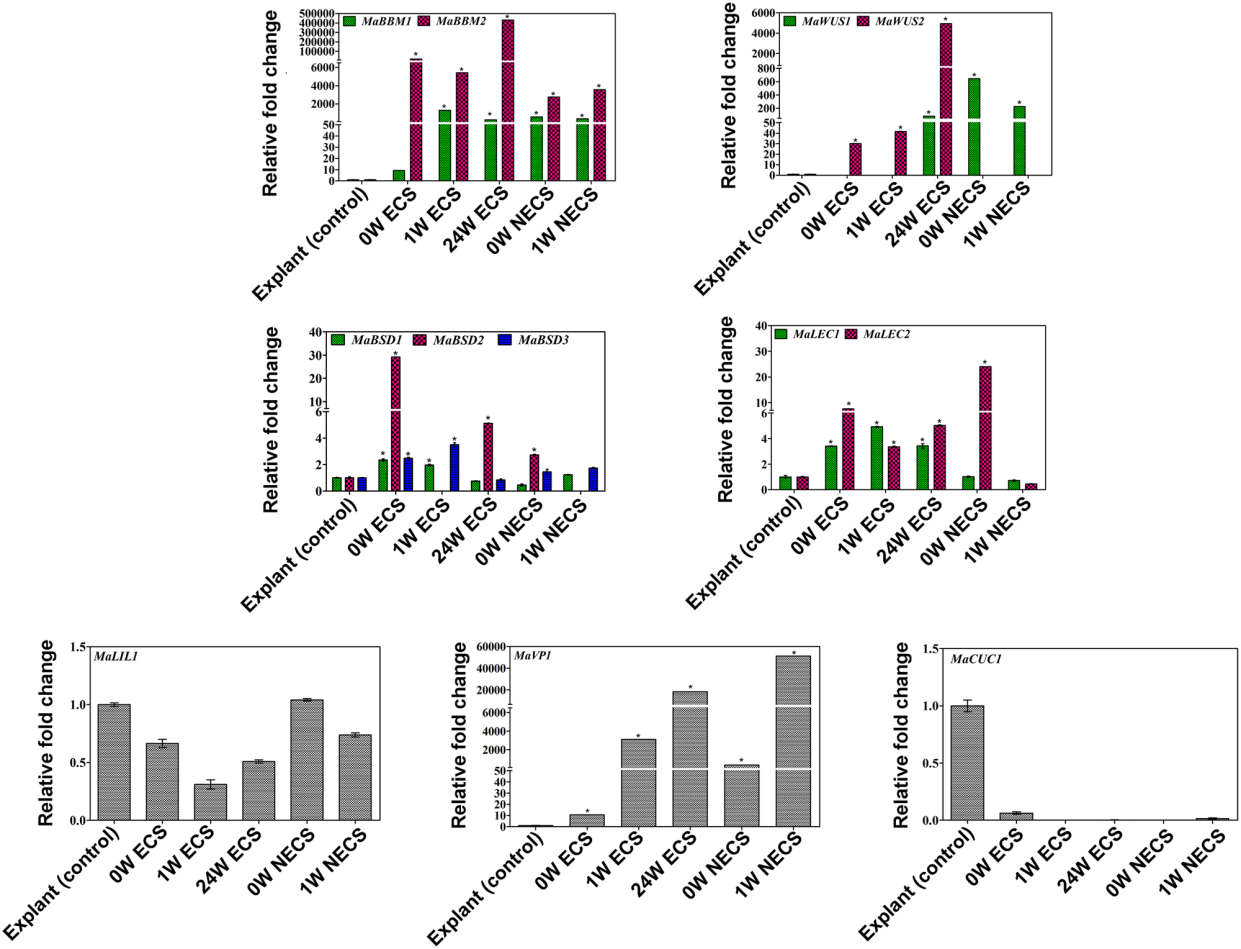
Quantitative real-time PCR study of the selected transcription factors in ECS. Differential expression of selected TFs in different development stages of ECS (0W, 1W, and 24W) and NECS (0W and 1W). The gene expression was normalized using *Actin1* as an internal control. Bars denote mean fold expression as compared to the control (explant) ± SD. The color bars indicate the homolog of the respective gene. (* indicates p<0.05).

Differential expression of all 18 genes was observed at various developmental stages of ECS (0W, 1W, and 24W) and NECS (0W and 1W) cultures (Fig 7). The male flower (explant) was used as a control for the expression study. *MaBBM1*, *MaBBM2*, *MaWUS1*, *MaLEC2*, and *MaVP1* were expressed differentially in 0W and 1W NECS. The higher expression of *MaBBM* (*MaBBM1* and *MaBBM2*) was noticed in both ECS and NECS. However, *MaBBM2* showed increased transcript level in comparison to *MaBBM1* in both ECS and NECS (Fig 7). The expression of *MaWUS2* was gradually increased in different stages of ECS, but it was undetected in NECS. In contrast, *MaWUS1* was highly expressed in different stages of NECS, but in the case of ECS, its expression was detected only at 24W. Out of three paralogs of *MaBSD*, the expression of *MaBSD2* was significantly upregulated at early (0W) and late (24W) stages of ECS, while *MaBSD3* showed higher expression at 1W ECS. *MaBSD2* and *MaBSD3* were expressed only at 0W and 1W NECS, respectively.

The higher expression of *MaLEC2* was noticed in all stages of ECS and 0W NECS. On the other side, *MaLEC1* showed higher expression at different stages of ECS but not NECS. *MaLEC2* was highly expressed at 0W NECS. *MaVP1* in banana was significantly upregulated at different development stages of ECS and NECS. *MaL1L1* and *MaCUC1* did not show any significant upregulation in expression level as compared to the explant. The expression of *MaL1L2*, *MaCUC2*, *MaCUC3*, *MaAGL*, and *MaBOL* was not detected in the qPCR study.

## Discussion

SE is widely utilized for micropropagation and genetic transformation, but the basic molecular mechanism behind it is not well understood [58–59]. Recently, the proteome approach has been reported to identify the differentially expressed proteins during the SE in banana [2]. Gene expression study of SE related TFs could be the significant approach for understanding their role in *in*-*vitro* developmental biology. In banana potency of explant to develop embryogenic callus is very low [59]. Moreover, the prolonged culturing in the callus induction medium could also lead to a somaclonal variation [11]. Therefore, it is important to find the molecular regulators that can be explored to enhance the SE potential in banana. In this study, *in-silico* characterization and expression analysis of the 18 genes of 9 TF families were studied for their role in SE of banana. Homologs of most of these genes in other plant species have already been reported for their role in SE [30–31, 15, 38, 60, 29, 25, 61]. The presence of multiple homologs of TFs in banana may be the result of gene duplication events during evolution, which may have significance for functional divergence [53].

The differential expression patterns of 18 genes in embryogenic and non-embryogenic tissues, and cell suspension cultures with respect to the explant of cv. Grand Naine were determined. *MaBBM2* is highly expressed in all stages of ECS. The expression of *MaBBM2* in ECS was increased to 22.95 fold at 24W as compared to 0W. However, the lower expression of *MaBBM2* was noticed in NECS as compared to ECS. *BBM* role in the conversion of vegetative tissue to embryogenic culture has been reported in *Brassica napus* [15]. In arabidopsis, overexpression of *GmBBM1* resulted in somatic embryos emergence in transgenic lines [62]. Similarly, our expression study also revealed that *MaBBM2* may play an important role in the conversion of explant to embryogenic callus and ECS.

*WUS* contains a homeodomain that is involved in regulation of developmental processes [21, 63]. *WUS* role in promoting vegetative to embryogenic transition and stem cell maintenance has been reported in arabidopsis [21]. Among the two homologs of *WUS* under study, *MaWUS2* was found to be highly expressed in the late stage of ECS that suggests its potential role in ECS maintenance. Recently, *BBM* and *WUS* have also been reported to improve transformation efficiency in monocots i.e., sorghum, sugarcane and rice [64].

BSD domain containing genes are known to be a basal TF which are reported for their association with cell proliferation during SE [34]. Here, we identified the three homologs of *BSD* (*MaBSD1*, *MaBSD2*, and *MaBSD3*) in banana. MaBSD homologs shared two distinct clades in phylogenetic tree analysis. MaBSD2 clustered with gymnosperm, while MaBSD1 and MaBSD3 were grouped in a clade with monocot. This indicates their divergence during the evolution. *MaBSD2* was differentially expressed in ECS, whereas *MaBDS1* did not show any significant change in expression level. These results suggested that gene duplication during the evolution process may hamper the functionality of *MaBSD* homologs.

*LEC* plays role in controlling embryogenesis [65]. The present study showed high accumulation of *MaLEC2* transcript in 0W NECS. It suggests that *MaLEC2* could lead to the cell necrosis via non-embryogenic callus in banana. However, *LEC1* was expressed in embryogenic cells as compared to non-embryogenic cells in carrot [66]. The L1L has close structural resemblance with LEC and known to be a regulator of embryo development. In our study, the homologs of *MaL1L* did not show any significant change in the expression.

*VP1* an auxin-inducible gene that encodes a TF involved in ABA signaling [67–68]. We noticed that *MaVP1* was highly expressed in callus, ECS, and NECS. Expression of *VP1* in ECS of arabidopsis has been correlated with its role in embryo development [38]. In *Secale cereale*, *VP1* has been reported to have a negative effect on the development of embryogenic callus [69].

*CUC* is known to induce adventitious shoots in arabidopsis [70–72]. It is utilized as a predictive marker for root and shoot organogenesis [39, 73]. Hence, we selected *CUC* gene from banana as a negative control for the expression study. As expected *CUC* homologs were either absent or having basal expression level in different development stages of SE in banana. The expression of *MaAGL* and *MaBOL* was not detected. Previous reports showed *AGL15* accumulation in tissues derived from double fertilization and its participation in the early stages of zygotic embryo development [33]. The significantly higher expression of *MaLEC2* at 0W NECS has suggested their role towards non-embryogenecity in banana.

Based on the differential expression patterns, we anticipate that *MaBBM2* and *MaWUS2* are the promising candidates for the embryogenicity in banana. Besides, it would be needed to confirm the functional role of *MaLEC2* in the development of non-embryogenic callus. Therefore, future studies will be directed to functionally assess the role of *MaBBM2*, *MaWUS2*, and *MaLEC2* for the better understanding of SE in banana.

## Supporting information

S1 Fig Multiple sequence alignment of MaBBM transcription factor with its other homologs. Amino acid sequences with two repeats of the conserved AP2 domain are highlighted. Homologs selected for the study, BnBBM1 (*Brassica napus* accession no. AF317904), BnBBM2 (*Brassica napus* accession no. AF317905), AtBBM (*Arabidopsis thaliana* accession no. NP_197245), GmBBM (*Glycine max* accession no. HM775856).
(TIF)

S2 Fig Multiple sequence alignment of MaWUS transcription factor with other homologs. Amino acid sequences with conserved Homeodomain are highlighted. Homologs selected for the study, VvWUS (*Vitus vinifera* accession no. XP_002266323.1), AtWUS1 (*Arabidopsis thaliana* accession no.NP_565429.1), OsWUS (*Oryzasativa* accession no.AB218894), PaWUS (*Picea abies* accession no. JX512364), AtWUS2 (*Arabidopsis thaliana* accession no. NM_125325).
(TIF)

S3 Fig Multiple sequence alignment of aMaBSD transcription factor with other homologs. Amino acid sequences with the conserved domain (BSD) are highlighted. Homologs selected for the study, PtBSD (*Populus trichocarpa* accession no. XP_002310888), VvBSD (*Vitis vinifera* accession no. XP_010647076), ZomBSD (*Zostera marina* accession no. KMZ65018), PsBSD (*Picea sitchensis* accession no. ABK24663).
(TIF)

S4 Fig Multiple sequence alignment of MaLEC/L1L transcription factor with other homologs. Amino acid sequences with the conserved HAP3-B domain are highlighted. Homologs selected for the study, GlLEC (*Glycine latifolia* accession no. ABW7151), ZmLEC (*Zea mays* accession no. AF410176), PcLEC (*Pinus contorta* accession no. HM852975) AtLEC (*Arabidopsis thaliana* accession no. NP_173616), PdL1L (*Phoenix dactylifera* accession no.XP_008786198), ZmL1L (*Zea mays* accession no. NP_001167647), PsL1L (*Picea sitchensis* accession no. ABK25387).
(TIF)

S5 Fig Multiple sequence alignment of MaVP1 transcription factor with other homologs. Amino acid sequences with the conserved domain (acidic and basic regions) are highlighted. Homologs selected for the study, NnVP1 (*Nelumbo nucifera* accession no. XP_010245692), VvVP1 (*Vitis vinifera* accession no. XP_003632397), ZmVP1 (*Zea mays* accession no. NM_001112070), PaVP1 (*Picea abies* accession no. AAG22585).
(TIF)

S6 Fig Multiple sequence alignment of MaCUC transcription factor with other homologs. Amino acid sequences with the conserved domain (NAC) are highlighted. Homologs selected for the study, EgCUC (*Elaeis guineens* accession no. HM62227) PsCUC (*Picea* sitchensis accession no. ABR16679), CpCUC (*Carica papaya* accession no. BK007973), PaCUC (*Picea abies* accession no. ADQ47506), PsCUC (*Picea sitchensis* accession no. ABR16679).
(TIF)

S7 Fig Multiple sequence alignment of MaBOL transcription factor with other homologs. Amino acid sequences with the conserved domain (BOLA) are highlighted. JrBOL (*Juglans regia* accession no. XP_018860422), CsBOL (*Cucumis sativus* accession no. XP_004147354), PdBOL (*Phoenix dactylifera* accession no.XP_008785827), PsBOL (*Picea sitchensis* accession no. ABK22779).
(TIF)

S8 Fig Multiple sequence alignment of MaAGL transcription factor with other homologs. Amino acid sequences with the conserved domain (MADS domain) are highlighted. Homologs selected for the study, AtAGL (*Arabidopsis thaliana* accession no. AAB3897), VvAGL (*Vitis vinifera* accession no. AF373603), GbAGL (*Ginkgo biloba* accession no. AAM76208), EgAGL (*Elaeis guineensis* accession no. XP_01090993).
(TIF)

S1 Table Primers used for real-time PCR study. (DOCX)

S2 Table Percentage similarity of the predicted protein sequences of *Musa acuminata* with other plant spceies. (DOCX)

S3 Table The exon-intron prediction of *Musa acuminata*, *Arabidopsis thaliana*, *Zea mays* and *Oriza sativa* homologs. (DOCX)
