# Supplementary figures and images for: Genome-wide analysis of transcription factors during somatic embryogenesis in banana (Musa spp.) cv. Grand Naine
Source: PLoS One. 2017 Aug 10;12(8):e0182242. doi: 10.1371/journal.pone.0182242 (PMC5552287; doi:10.1371/journal.pone.0182242)

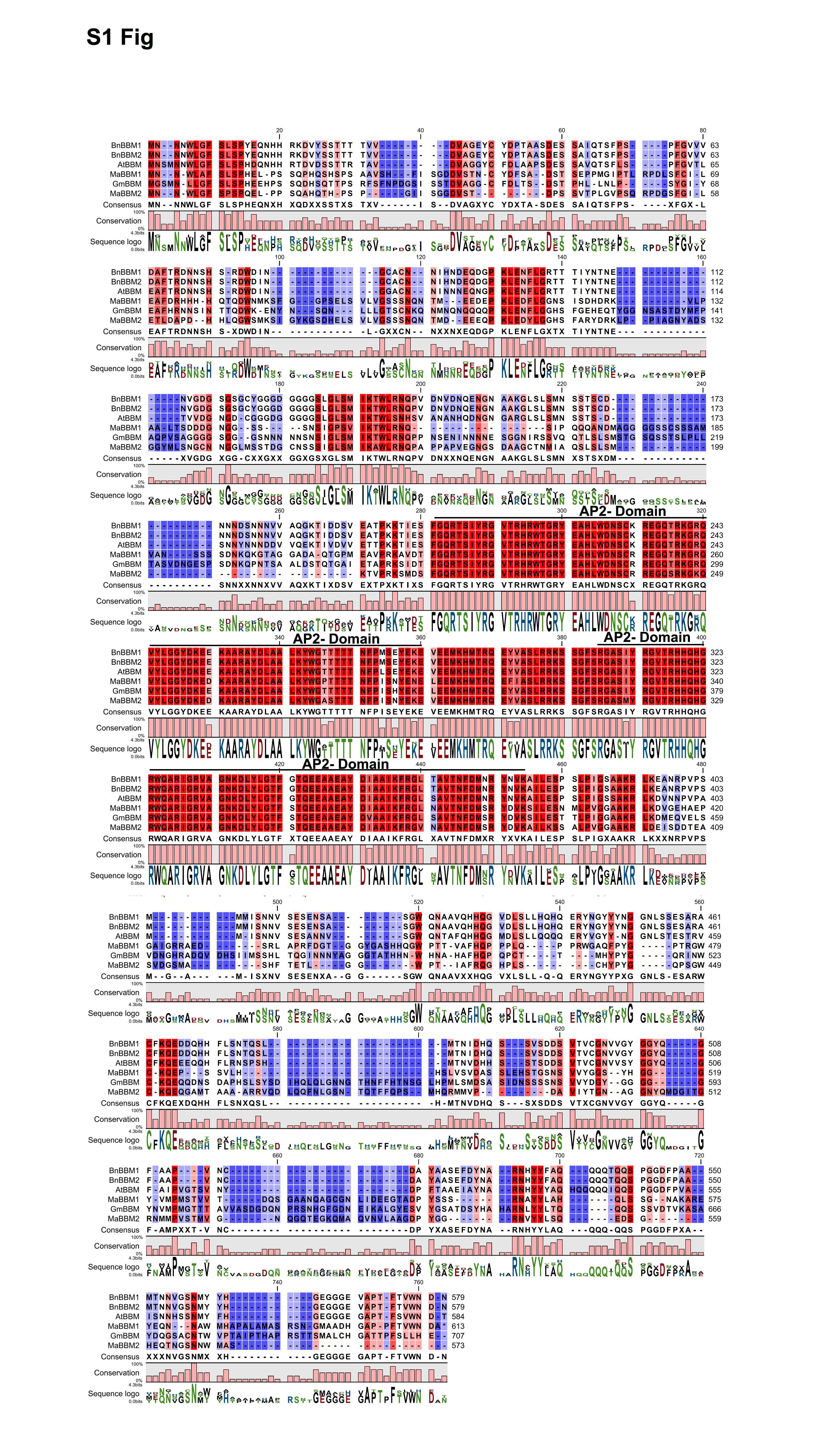

Supplement: S1 Fig — Amino acid sequences with two repeats of the conserved AP2 domain are highlighted. Homologs selected for the study, BnBBM1 (Brassica napus accession no. AF317904), BnBBM2 (Brassica napus accession no. AF317905), AtBBM (Arabidopsis thaliana accession no. NP_197245), GmBBM (Glycine max accession no. HM775856). (TIF) [file pone.0182242.s001.tif]

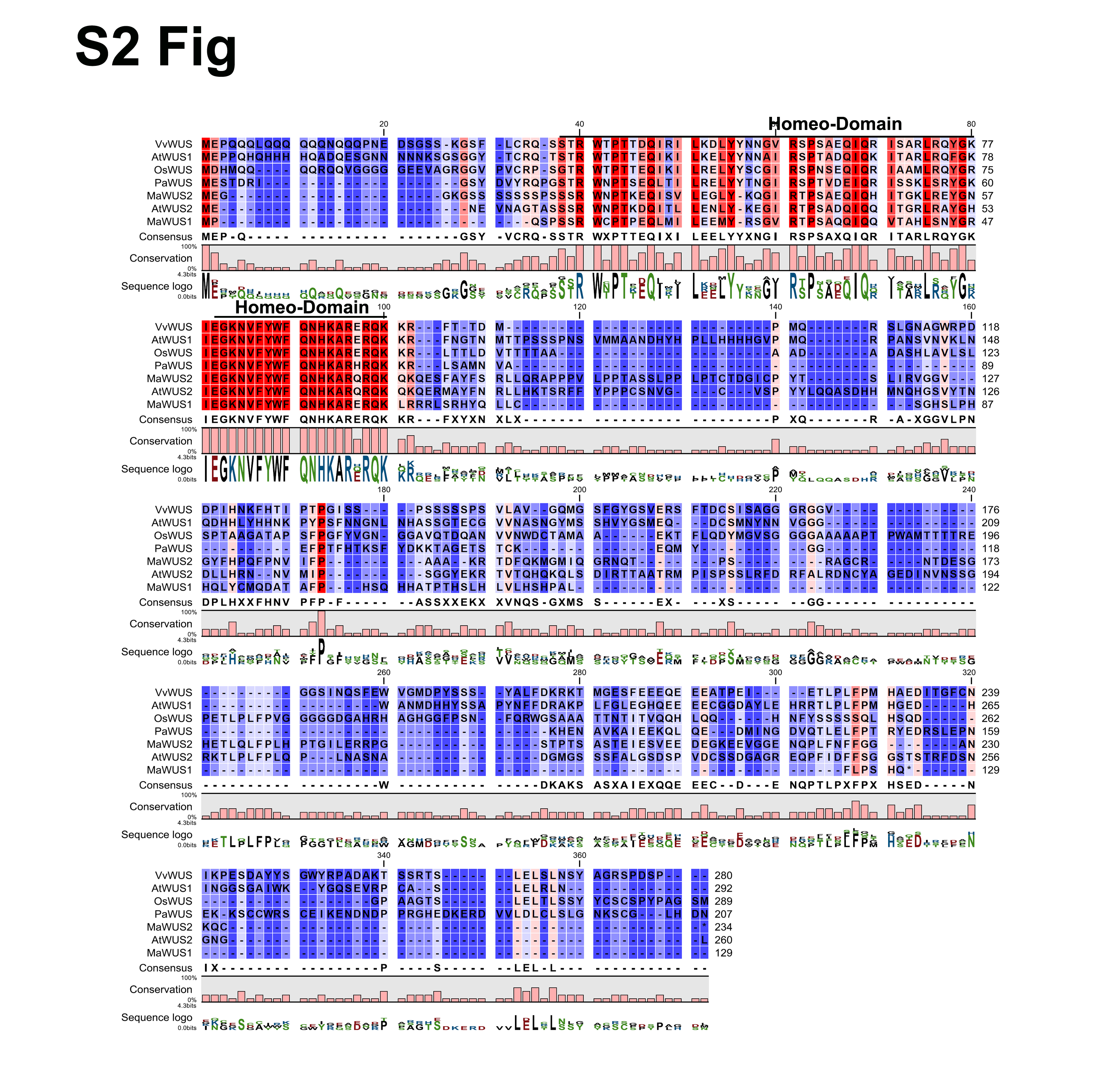

Supplement: S2 Fig — Amino acid sequences with conserved Homeodomain are highlighted. Homologs selected for the study, VvWUS (Vitus vinifera accession no. XP_002266323.1), AtWUS1 (Arabidopsis thaliana accession no.NP_565429.1), OsWUS (Oryzasativa accession no.AB218894), PaWUS (Picea abies accession no. JX512364), AtWUS2 (Arabidopsis thaliana accession no. NM_125325). (TIF) [file pone.0182242.s002.tif]

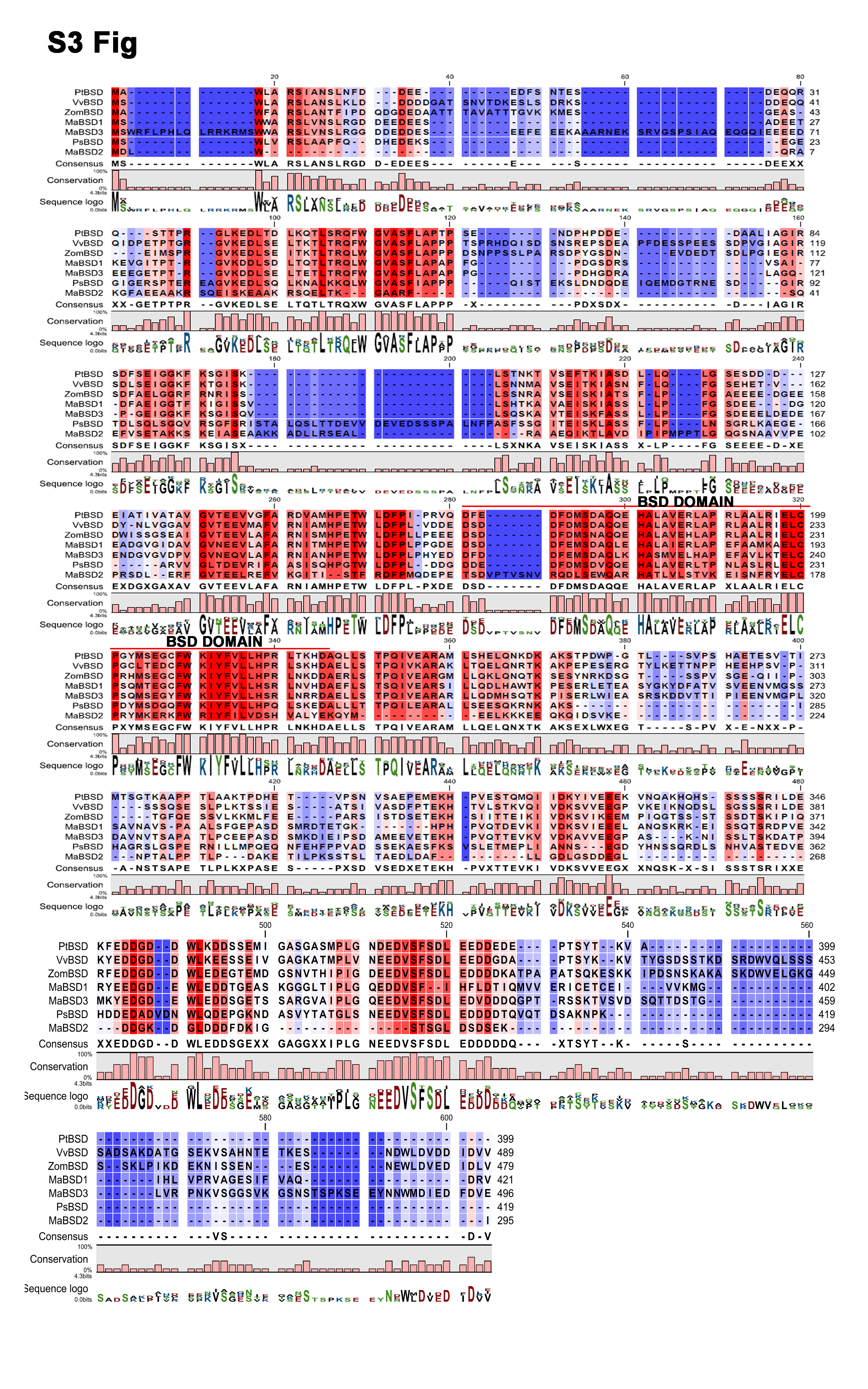

Supplement: S3 Fig — Amino acid sequences with the conserved domain (BSD) are highlighted. Homologs selected for the study, PtBSD (Populus trichocarpa accession no. XP_002310888), VvBSD (Vitis vinifera accession no. XP_010647076), ZomBSD (Zostera marina accession no. KMZ65018), PsBSD (Picea sitchensis accession no. ABK24663). (TIF) [file pone.0182242.s003.tif]

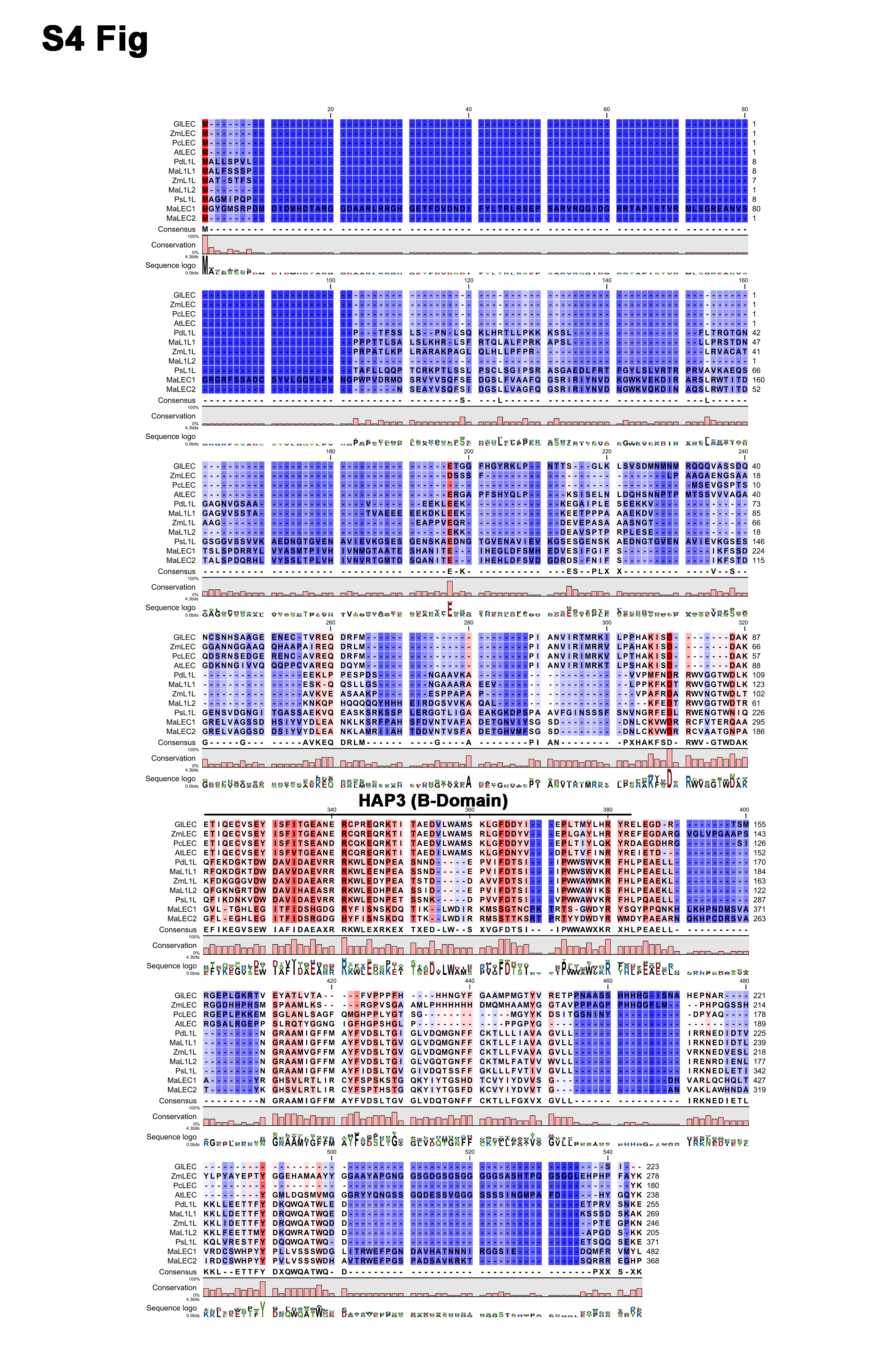

Supplement: S4 Fig — Amino acid sequences with the conserved HAP3-B domain are highlighted. Homologs selected for the study, GlLEC (Glycine latifolia accession no. ABW7151), ZmLEC (Zea mays accession no. AF410176), PcLEC (Pinus contorta accession no. HM852975) AtLEC (Arabidopsis thaliana accession no. NP_173616), PdL1L (Phoenix dactylifera accession no.XP_008786198), ZmL1L (Zea mays accession no. NP_001167647), PsL1L (Picea sitchensis accession no. ABK25387). (TIF) [file pone.0182242.s004.tif]

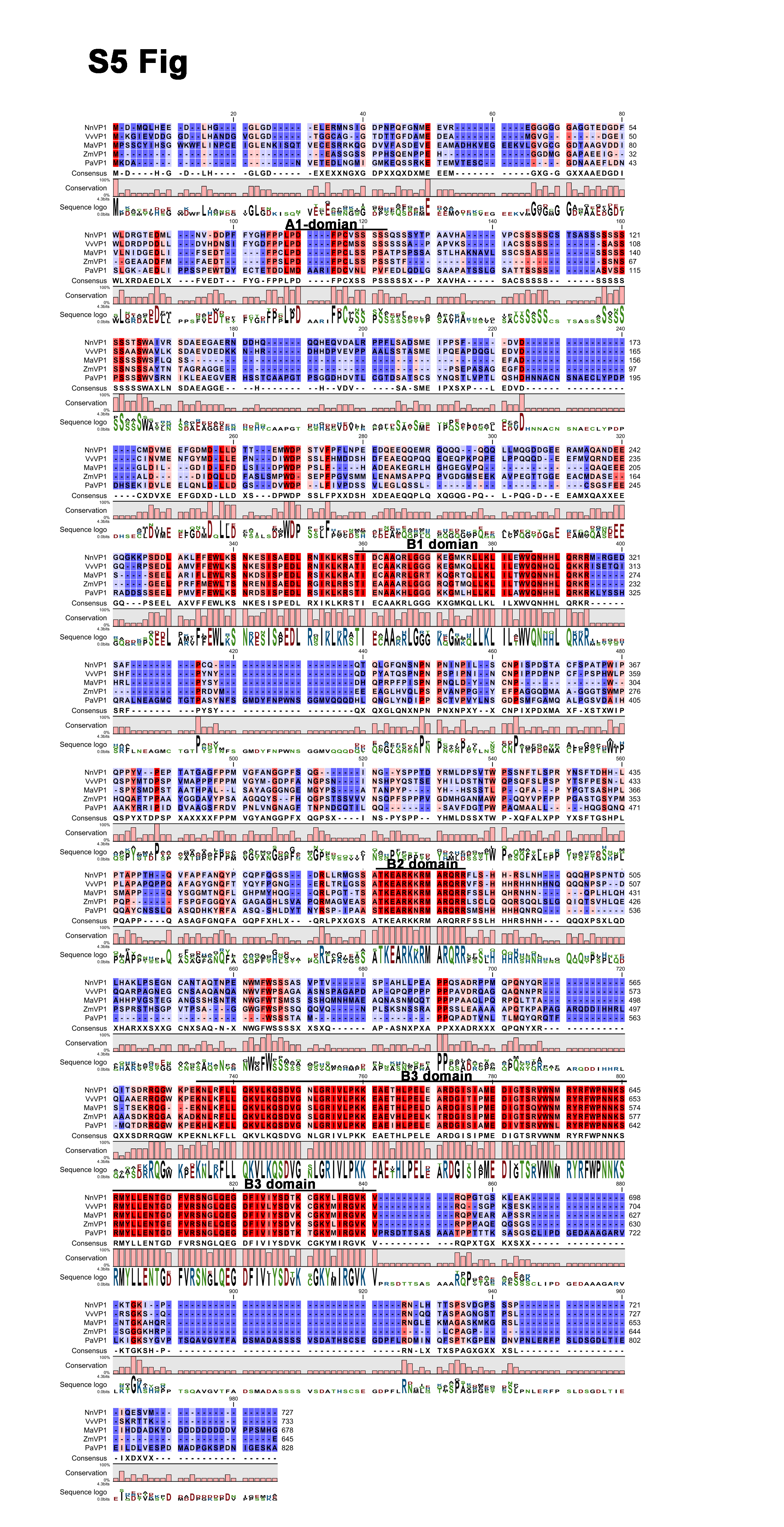

Supplement: S5 Fig — Amino acid sequences with the conserved domain (acidic and basic regions) are highlighted. Homologs selected for the study, NnVP1 (Nelumbo nucifera accession no. XP_010245692), VvVP1 (Vitis vinifera accession no. XP_003632397), ZmVP1 (Zea mays accession no. NM_001112070), PaVP1 (Picea abies accession no. AAG22585). (TIF) [file pone.0182242.s005.tif]

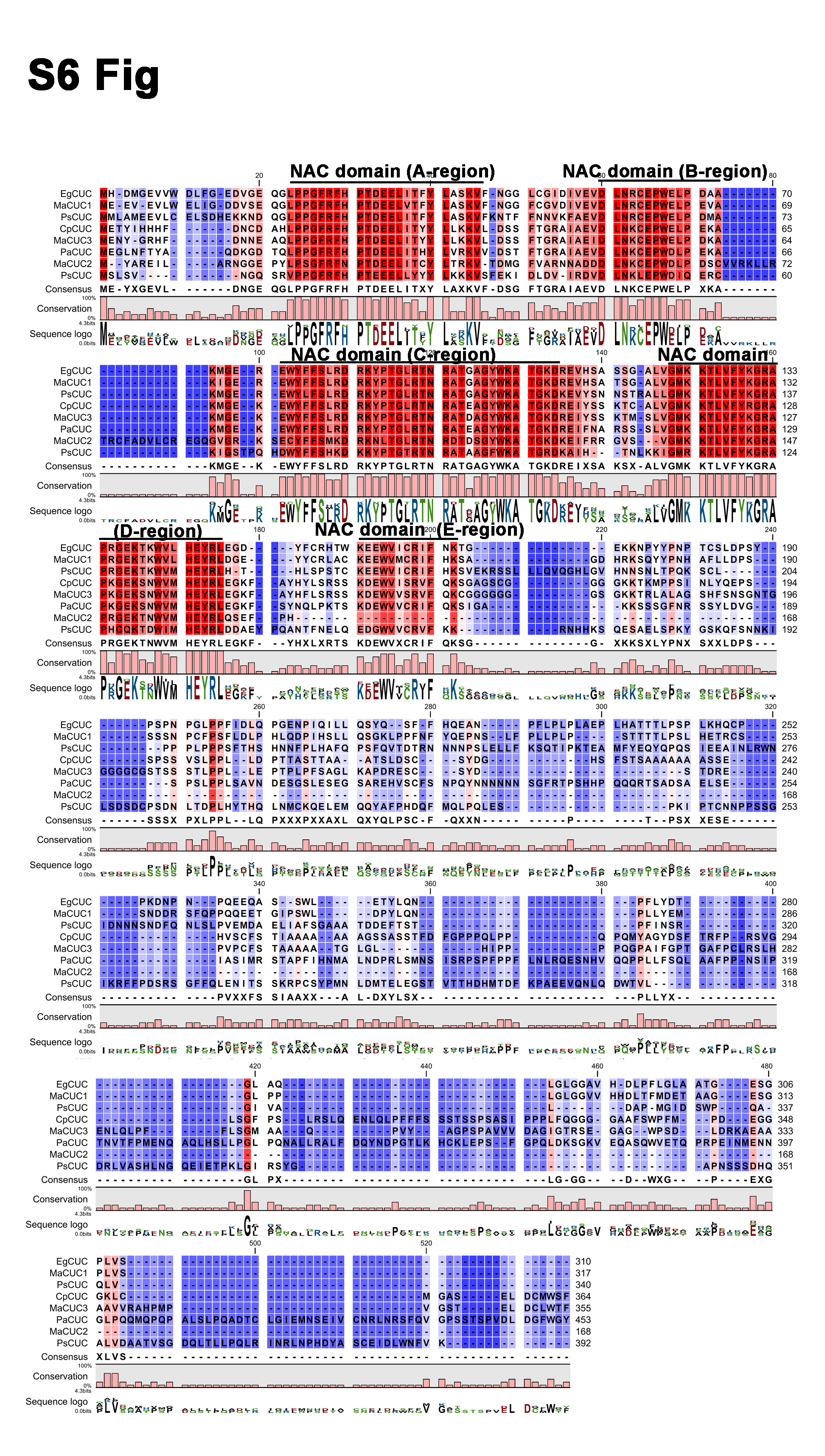

Supplement: S6 Fig — Amino acid sequences with the conserved domain (NAC) are highlighted. Homologs selected for the study, EgCUC (Elaeis guineens accession no. HM62227) PsCUC (Picea sitchensis accession no. ABR16679), CpCUC (Carica papaya accession no. BK007973), PaCUC (Picea abies accession no. ADQ47506), PsCUC (Picea sitchensis accession no. ABR16679). (TIF) [file pone.0182242.s006.tif]

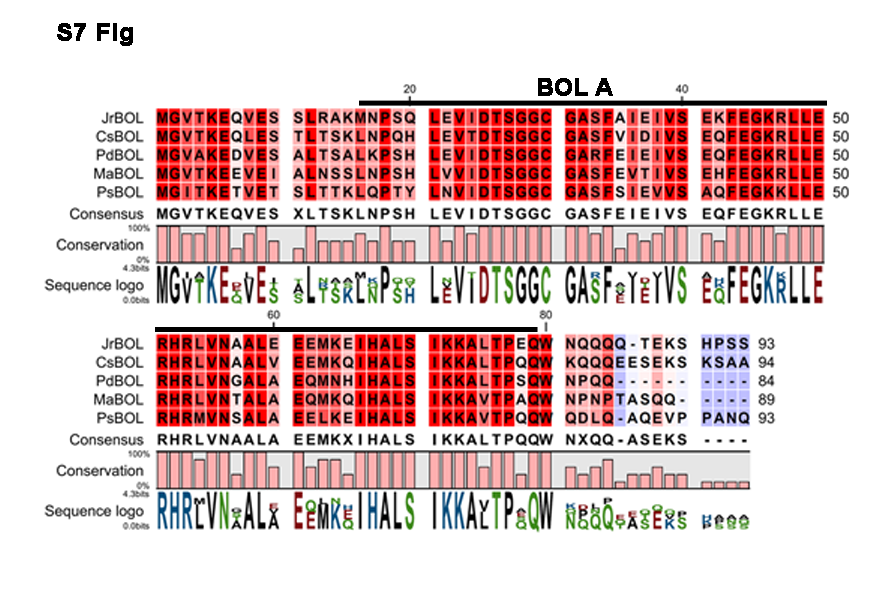

Supplement: S7 Fig — Amino acid sequences with the conserved domain (BOLA) are highlighted. JrBOL (Juglans regia accession no. XP_018860422), CsBOL (Cucumis sativus accession no. XP_004147354), PdBOL (Phoenix dactylifera accession no.XP_008785827), PsBOL (Picea sitchensis accession no. ABK22779). (TIF) [file pone.0182242.s007.tif]

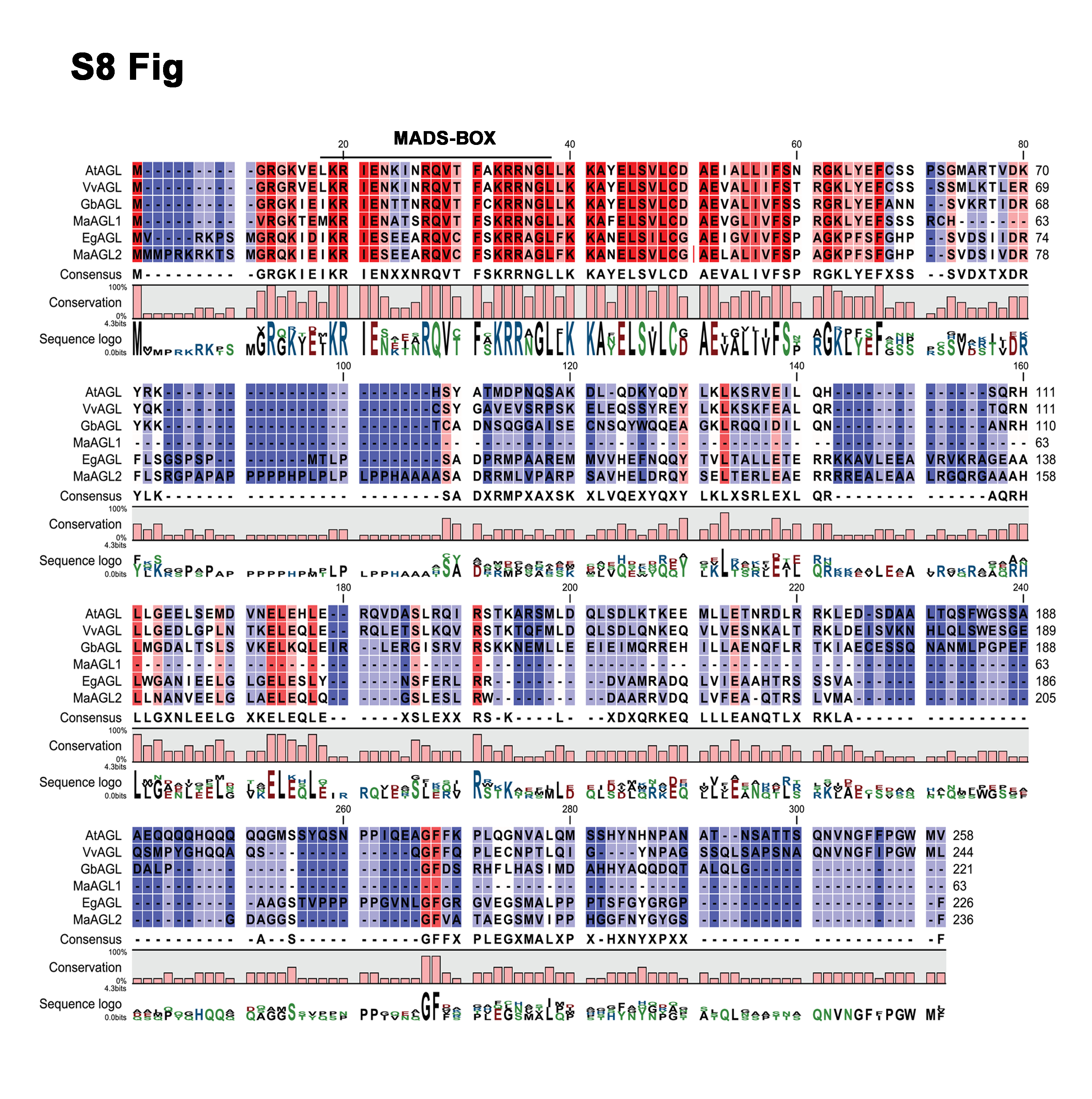

Supplement: S8 Fig — Amino acid sequences with the conserved domain (MADS domain) are highlighted. Homologs selected for the study, AtAGL (Arabidopsis thaliana accession no. AAB3897), VvAGL (Vitis vinifera accession no. AF373603), GbAGL (Ginkgo biloba accession no. AAM76208), EgAGL (Elaeis guineensis accession no. XP_01090993). (TIF) [file pone.0182242.s008.tif]
