# Supplementary material for: Genome-wide analysis of transcription factors during somatic embryogenesis in banana (Musa spp.) cv. Grand Naine
Source: PLoS One. 2017 Aug 10;12(8):e0182242. doi: 10.1371/journal.pone.0182242 (PMC5552287; doi:10.1371/journal.pone.0182242)
Supplement: S1 Table — (DOCX) [file pone.0182242.s009.docx]

**S1 Table.** **Primers used for real-time PCR study.**

| **Primer Name** | **Forward Sequence (5'-3')** | **Reverse Sequence (5'-3')** |
| --- | --- | --- |
| *MaBBM1* | TCGCAGGGAACAAAGACCTC | GTACCGGCTCATGTCGAAGT |
| *MaBBM2* | AGGCCTACGACATTGCGGCA | GCTGCCATCAACACTTGCCTCTG |
| *MaWUS1* | CCAGATCTCGGAGACGAACG | GTTCAGGGATTCGACCTCGG |
| *MaWUS2* | GACCCCAAGTGCTCAACAGA | TTGGTAGTGCCTGCTAAGCC |
| *MaBSD1* | TGGATCGGACGAAGAGGAGT | TGGGGCAAAGGGAAATCCAG |
| *MaBSD2* | CGATTTTGCGGAGATCGGTG | CCTCCTCGTCCGATCCAAAC |
| *MaBSD3* | ATGCAAGATGAGCCGGAGAC | TCGTGGGCACAGTTCATACC |
| *MaLEC1* | CTGCATTCCAGGGAAGTCGT | GTCGATCTGGGGAGAGGGAT |
| *MaLEC2* | GACCGGGATGACAGACTCAC | CAAGCTCTCGACCATCGGTT |
| *MaLIL1* | AGCGGTTTCAGAAGGATGGG | ACAGGCTCGTCATTGCTTGA |
| *MaLIL2* | AGGCCGTTTCTCCTACTCCT | CCTTCACCACGGAACCATCA |
| *MaVP1* | GGGGAGTGAAGGTTCGACAG | TCATTTTTGATGCGCCAGCC |
| *MaCUC1* | AACAAGTGCGAACCTTGGGA | CTTCCCAGTTGCCTTCCAGT |
| *MaCUC2* | TGCTATTTGACCCGCAAGGT | TTACTACGCATGAGTCCGGC |
| *MaCUC3* | AGCCTCCAAGGTCTTCAACG | TATCTTCGCCACCTCTGGGA |
| *MaBOL* | AGTGACTATCGTGTCGGAGC | GGCGTGGATCTGCTTCATCT |
| *MaAGL1* | GGTGACATTCTCCAAGCGGA | TTCCCTCGGGGAGAGAAGAC |
| *MaAGL2* | GTGGATCAGCTGGTGTTCGAG | ACCGTGAGGAGGAATCACCA |
| *MaActin1* | ATGACATGGAGAAGATATGGCATCA | AGCCTGGATGGCAACATACATAGC |
