# Supplementary material for: Genome-wide analysis of transcription factors during somatic embryogenesis in banana (Musa spp.) cv. Grand Naine
Source: PLoS One. 2017 Aug 10;12(8):e0182242. doi: 10.1371/journal.pone.0182242 (PMC5552287; doi:10.1371/journal.pone.0182242)
Supplement: S2 Table — (DOCX) [file pone.0182242.s010.docx]

**S2 Table. Percentage similarity of the predicted protein sequences of *Musa acuminata* with other plant species.**

| **Gene** | ***Musa acuminata*** | ***Arabidopsis thaliana*** | ***Zea mays*** | ***Oryza sativa*** |
| --- | --- | --- | --- | --- |
|  |  |  |  |  |
| *MaBBM1* | GSMUA_Achr3P21460_001 | AT5G17430.1 46% | 0 | 0 |
| *MaBBM2* | GSMUA_Achr2P05880_001 | AT5G17430.1 46% | 0 | 0 |
| *MaWUS1* | GSMUA_Achr10P26570_001 | AT3G15880.1 67% AT3G15880.2 67% AT3G15880.3 67% AT2G17950.1 65% | ZM02G01420 67%  ZM10G25800 66%  ZM06G04160 65% ZM03G26590 60% ZM06G31190 46% ZM08G31360 46% | 0 |
| *MaWUS2* | GSMUA_Achr8P02040_001 | AT2G17950.1 65%  AT1G03170.1 35% | ZM02G24320 68% ZM02G01420 65% ZM10G25800 65% ZM06G04160 64% ZM03G26590 61% ZM06G31190 46% ZM08G31360 46% | 0 |
| *MaBSD1* | GSMUA_Achr2P17230_001 | AT1G55750.1 53%  AT1G03350.1 48%  AT3G61420.1 47%  AT3G49800.1 40%  AT1G10720.1 39%  AT2G10950.1 38%  AT1G26300.2 38%  AT5G65910.1 36% | ZM08G34220 42%  ZM01G51650 36% | 0 |
| *MaBSD2* | GSMUA_Achr6P00640_001 | AT3G61420.1 53% AT5G65910.1 42% AT2G10950.1 40% AT3G49800.1 39% AT1G10720.1 39% AT4G13110.1 35% | ZM08G34220 43%  ZM01G51650 39%  ZM04G23270 25% | 0 |
| *MaBSD3* | GSMUA_Achr8P25810_001 | AT1G26300.2 52% AT1G69030.1 51% AT1G26300.1 50% AT4G13110.1 47% AT3G24820.1 47% AT3G61420.1 42% AT3G49800.1 36% AT1G10720.1 35% | ZM04G23270 61%  ZM01G51650 36%  ZM08G34220 34% | 0 |
| *MaLEC1* | GSMUA_Achr10P12560_001 | AT1G21970.1 29%  AT5G47670.1 24%  AT5G47670.2 24% | ZM08G16090 58% | 0 |
| *MaLEC2* | GSMUA_Achr3P23760_001 | AT1G21970.1 NS  AT5G47670.1 NS  AT5G47670.2 NS | ZM08G16090 67% | 0 |
| *MaLIL1* | GSMUA_Achr4P26330_001 | AT4G17600.1 58%  AT5G47110.1 55% | ZM04G40610 69% | 0 |
| *MaLIL2* | GSMUA_Achr11P06580_001 | AT5G47110.1 67% AT4G17600.1 60% | ZM04G40610 66% | 0 |
| *MaVP1* | GSMUA_AchrUn_randomP18520_001 | AT1G19220.1 55%  AT3G26790.1 54%  AT1G13260.1 34%  AT1G68840.1 34%  AT1G68840.2 34% | ZM03G23310 42% | 0 |
| *MaCUC1* | GSMUA_Achr10P22350_001 | AT3G15170.1 57%  AT3G15500.1 55%  AT5G53950.1 50% | ZM06G02790 54% | LOC_Os08g40030.1 69%  LOC_Os02g15340.1 55%  LOC_Os03g03540.1 54%  LOC_Os08g01330.1 52%  LOC_Os12g03040.1 52%  LOC_Os11g03300.1 52% |
| *MaCUC2* | GSMUA_Achr9P00570_001 | AT5G53950.1 58%  AT3G15170.1 53%  AT3G15500.1 50% | Z0M06G02790 57% | LOC_Os08g40030.1 53%  LOC_Os03g03540.1 49%  LOC_Os02g15340.1 48%  LOC_Os02g06950.1 48%  LOC_Os08g01330.1 47%  LOC_Os12g03040.1 45% |
| *MaCUC3* | GSMUA_Achr9P20090_001 | AT5G53950.1 69%  AT3G15500.1 50%  AT3G15170.1 64% | ZM06G02790 70% | LOC_Os03g03540.1 57%  LOC_Os08g01330.1 56%  LOC_Os02g15340.1 52%  LOC_Os08g40030.1 51%  LOC_Os12g03040.1 49%  LOC_Os11g03300.1 49%  LOC_Os02g06950.1 47% |
| *MaBOL* | GSMUA_Achr6P19570_001 | 0 | ZM02G13720 74% | LOC_Os01g64680.1 74% |
| *MaAGL1* | GSMUA_Achr5P20280_001 | AT5G13790.1 78 % AT3G57390.1 73 % | 0 | 0 |
| *MaAGL2* | GSMUA_Achr8P07230_001 | AT1G79760.1 83%  AT5G13790.1 59% | 0 | 0 |

* NS- Not Significant
