# Supplementary material for: Genome-wide analysis of transcription factors during somatic embryogenesis in banana (Musa spp.) cv. Grand Naine
Source: PLoS One. 2017 Aug 10;12(8):e0182242. doi: 10.1371/journal.pone.0182242 (PMC5552287; doi:10.1371/journal.pone.0182242)
Supplement: S3 Table — (DOCX) [file pone.0182242.s011.docx]

**S3 Table. The exon-intron prediction of *Musa acuminata*, *Arabidopsis thaliana*, *Zea mays* and *Oryza sativa* homologs.**

| **Gene** | ***Musa acuminata*** | **Exon** | **Intron** | ***Arabidopsis thaliana*** | **Exon** | **Intron** | ***Zea mays*** | **Exon** | **Intron** | ***Oryza sativa*** | **Exon** | **Intron** |
| --- | --- | --- | --- | --- | --- | --- | --- | --- | --- | --- | --- | --- |
| ***MaBBM*** | GSMUA_Achr3P21460_001 GSMUA_Achr2P05880_001 | 12  10 | 11  09 | AT5G17430.1 | 09 | 08 | - |  |  | - |  |  |
| ***MaWUS*** | GSMUA_Achr8T02040_001  GSMUA_Achr10T26570_001 | 01  02 | 00  01 | AT2G17950.1  AT3G15880.1  AT3G15880.2  AT3G15880.3 | 03  25  26  25 | 02  24  25  24 | ZM02G01420  ZM03G26590  ZM03G27360  ZM06G04160  ZM06G31190  ZM10G25800 | 03  02  02  02  04  03 | 02  01  01  01  03  02 |  |  |  |
| ***MaBSD*** | GSMUA_Achr6T00640_001  GSMUA_Achr8T25810_001  GSMUA_Achr2T17230_001 | 07  06  05 | 06  05  04 | AT1G10720.1  AT1G03350.1  AT1G55750.1  AT5G65910.1  AT3G61420.1  AT3G49800.1  AT2G10950.1  AT3G24820.1 | 03  01  20  03  20  03  03  04 | 02  00  19  02  19  02  02  03 | ZM08G34220 ZM01G51650 | 03  01 | 02  00 | - |  |  |
| ***MaLEC*** | GSMUA_Achr10T12560_001  GSMUA_Achr3T23760_001 | 09  09 | 08  08 | AT1G21970.1  AT5G47670.1  AT5G47670.2 | 02  01  01 | 01  00  00 | ZM08G16090 | 11 | 10 |  |  |  |
| ***MaLIL*** | GSMUA_Achr4T26330_001  GSMUA_Achr11P06580_001 | 03  03 | 02  02 | AT4G17600.1  AT5G47110.1 | 03  03 | 02  02 | ZM04G40610 | 03 | 02 |  |  |  |
| ***MaVP1*** | GSMUA_AchrUn_randomT18520_001 | 09 | 08 | AT1G13260.1  AT1G68840.1  AT1G68840.2 | 01  01  01 | 00  00  00 | ZM03G23310 | 06 | 05 |  |  |  |
| ***MaCUC*** | GSMUA_Achr10P22350_001  GSMUA_Achr9T20090_001  GSMUA_Achr9T00570_001 | 03  03  02 | 02  02  01 | AT5G53950.1  AT3G15170.1  AT3G15500.1 | 03  03  03 | 02  02  02 | ZM06G02790 | 03 | 02 | Os08g40030  Os02g15340  Os02g06950  Os08g01330  Os12g03040  Os03g03540  Os11g03300 | 02  03  06  03  03  03  03 | 01  02  05  02  02  02  02 |
| ***MaBOL*** | GSMUA_Achr6T19570_001 | 03 | 02 |  |  |  | ZM02G13720 | 04 | 03 | Os01g64680 | 03 | 02 |
| ***MaAGL*** | GSMUA_Achr5T20280_001  GSMUA_Achr8T07230_001 | 01  01 | 00  00 | AT1G79760.1  AT3G57390.1  AT5G13790.1 | 02  08  08 | 02  07  07 | - |  |  | - |  |  |
